# DC vs. AC distribution: Revealing the efficiency advantage of DC in today’s energy landscape

**DOI:** 10.1371/journal.pone.0318444

**Published:** 2025-05-09

**Authors:** Asfand Haroon, Hasan Erteza Gelani, Hira Tahir, Habib Ullah Manzoor

**Affiliations:** 1 Department of Electrical Engineering, University of Engineering and Technology, Lahore, Pakistan; 2 Department of Energy Engineering, University of Engineering and Technology, New Campus, Kala Shah Kaku, Pakistan; 3 Interdisciplinary Research Center of Smart Mobility and Logistics, King Fahd University of Petroleum and Minerals, Dhahran, Saudi Arabia; 4 James Watt School of Engineering, University of Glasgow, Glasgow, United Kingdom; Public Library of Science, UNITED KINGDOM OF GREAT BRITAIN AND NORTHERN IRELAND

## Abstract

The new millennium witnessed an unanticipated escalation in the installation of rooftop solar panels, particularly due to the development of highly efficient power electronic converters (PECs). The ‘battle of currents’ between AC and DC, which settled in the favor of AC in the nineteenth century, reignited as DC is striking back due to this technological augmentation. The shifting trend towards DC is more pronounced in the residential sector, which necessitates a comparative analysis of AC and DC at distribution scale on realistic grounds. Modern home data extracted from the energy information administration (EIA) has been utilized to devise a mathematical model based on bottom-up approach. The comparative analysis has been performed encompassing scenarios of varying PEC efficiencies as a result of daily load variation. Moreover, the scenarios of multiple PEC efficiencies and rooftop solar capacities are also considered. The comparative analysis revealed efficiency advantage of 1.966%, 1.41% and 1.17% in favor of DC as compared to AC for the scenarios considered. In the end future recommendations are presented to further enhance the efficiency of DC, thereby providing a concrete standing for power industry decision of adopting DC at distribution scale.

## 1. Introduction

The birth of electricity, in nineteenth century, observed a rivalry that termed as ‘battle of currents’; between AC and DC. The battle was actually a conceptual rivalry between the two great minds of the time [1,2] Thomas Edison and Nikola Tesla with Edison advocating DC and Tesla advocating AC as medium of power transfer [3]. However, the battle did not last long and ended in favor of AC because of the revolutionary discovery of electromagnetic transformer by Tesla, capable of transforming voltage levels thereby making AC a better choice for long distance power transfer [4,5]. In contrast DC lost its credibility because there was no device available or invented at that time, for DC voltage transformation. AC was authenticated to be more efficient than DC for long distance power transfers due to its ability of voltage transformations. This highlights two important terms i-e efficiency and ability of voltage transformation; with efficiency as the parameter that wiped DC out of the power system and voltage transformation as the ability that AC possessed and made it superior than DC [6–10].

AC ruled the power system for decades, but its era was not everlasting. The field of power electronics made remarkable advancements and devised efficient power electronic converters (PECs) that possessed the ability of voltage transformations for DC [11,12], the same factor that DC lacked and lost the ‘battle of currents’. This invention of PECs reignited the battle and DC is striking back with the strength to conquer the power system. AC, which enjoyed supremacy in all the sectors of power; generation, transmission, distribution and utilization; is now getting weaker as compared to DC due to the invention of PECs [13,14]. In 1954, the world witnessed the first commercial high voltage DC line, Gotland 1, that proved to be better than AC and hence DC made its concrete standing in the sector of power transmission [15]. In the sector of generation, DC has made a firm foot with solar based generation, which is DC in nature [16,17]. Even wind can be better optimized with DC, with the reduction of a PEC stage [18,19]. The electronic devices in our homes such as laptops and mobile chargers, modern energy efficient appliances, almost every residential appliance is moving to DC side; making the utilization sector a success for DC [20–22]. The distribution sector is still under research [7–10]. A manifestation in this regard can be; when generation, transmission and utilization have moved to DC, why not distribution? This has to be proved on concrete grounds. Efficiency was the factor that once defined the victory of AC over DC, therefore, the same factor can be employed to define the comparison of AC and DC at distribution scale [23–25].

The efficiency analyses of AC and DC distribution systems has become a topic of interest for researchers with DC striking back in other sectors of power system. A number of authors have presented their research efforts in the relevant topic. However, most of the authors considered limited scenarios for the comparison or presented their work based on assumptions that diverted the analysis from reality. A comparative analysis demands closeness to reality by paying due consideration to the factors that affect overall system efficiency. Consideration of loads for the comparative analysis is an important factor. Authors of [26,27] considered a single type of load whereas authors of [28–32] considered a small set of selective loads. The authors of [26,27] presented the analysis ignoring the load variation throughout the day or actual home load profiles. In contrast the current paper has taken into account the data from energy information administration [33] of realistic US home. The current age witnessed the modern and energy efficiency variable speed drive (VSD) based loads which have been ignored by the authors of [34–38], whereas the current analysis pays due consideration to load classifications. The efficiency analysis is incomplete when the effect of varying efficiency of PECs with variation in attached loads is ignored. The authors of [34,39–46] assumed fixed PEC efficiencies or considered then within a specific range [47–49]. The power demand of loads within a residence varies continuously throughout the day. The efficiencies of installed PECs thereby vary with the load variation leading to varying system efficiency. Completely ignoring or considering fixed PECs efficiencies cannot address the situation and produce non-realistic results as in reality the loads keep varying, hence the PECs efficiencies leading to varying system efficiency. On the contrary, the current research effort addresses the situation by considering daily load variation, extracting the installed PECs efficiencies through the loading versus efficiency curve of PECs and employing the relevant efficiency in the analysis.

The current analysis is undoubtedly a realistic analysis that considers realistic home loads, with realistic classification and consideration of the effect of varying PECs efficiency in accordance with loading. The organization of the paper starts with system model, then a mathematical model using bottom-up approach for determining system efficiency, then analysis and concluding remarks.

## 2. System model

Data from EIA and Building Energy Outlook [33] for modern US based home is presented in Table 1. The table contains hourly power consumption (in Watt) of various loads for 24 hours in a US home. The loads have been classified into categories depending upon the power demand. In addition to the specifically presented significant loads, the term ‘other’ is used to address various small loads that may be turned ON and then OFF for specific use such as loads related to cosmetics, small kitchen appliances etc. The factor of fairness demands an equal proportion of such loads in each classification, which has been incorporated in the table and the analysis. The load classification is performed in the following fashion:

**Table 1:** Hourly power consumption in a day.

*VSD Loads (W)*					*DC Loads (W)*				*Unmediated Loads (W)*		
Space Cooling	Wet Cleaning	Refrige-ration	Space Heating	Other	Computer	Light	Elect-ronics	Other	Water Heating	Cooking	Other
183.75	113.6	35.365	91	81.81	164.954	47.67	82.68	81.81	73.8326	0	81.81
183.75	113.6	35.365	83	81.81	164.954	47.67	82.68	81.81	73.8326	0	81.81
122.5	109.813	16.752	80	81.81	164.954	47.67	84.054	81.81	60.7699	0	81.81
61.25	107.288	14.891	82	81.81	158.4	47.67	82.68	81.81	56.7943	0	81.81
0	107.288	35.365	87	81.81	151.85	47.67	82.68	81.81	73.833	0	81.81
0	108.551	37.226	89	81.81	140.92	47.67	82.66	81.81	90.871	14.2	81.81
0	108.551	44.6715	85	81.81	103.779	47.67	82.68	81.81	119.268	21.3	81.81
0	109.813	74.4525	105	81.81	102.69	47.67	84.054	81.81	159.024	63.9	81.81
0	112.338	83.7591	101	81.81	103.78	47.67	85.431	81.81	193.101	85.2	81.81
0	113.6	111.679	103	81.81	114.703	49.309	96.455	81.81	204.46	35.5	81.81
61.25	114.862	115.401	101	81.81	125.63	55.884	99.210	81.81	170.383	21.3	81.81
61.25	116.124	115.401	100	81.81	131.09	57.528	89.565	81.81	141.985	14.2	81.81
122.5	119.911	113.54	100	81.81	131.09	49.309	82.68	81.81	130.627	71	81.81
183.8	121.2	113.5	98	81.81	131.1	49.31	85.43	81.81	119.3	85	81.81
245	124.96	111.679	97	81.81	131.09	49.309	89.565	81.81	119.268	35.5	81.81
306.25	126.222	109.817	112	81.81	131.09	54.240	96.455	81.81	122.11	14.2	81.81
428.75	127.485	102.372	145	81.81	131.09	55.884	89.565	81.81	130.627	7.1	81.81
735	128.746	107.956	150	81.81	131.09	52.597	85.431	81.81	159.024	21.3	81.81
796.25	131.271	111.678	157	81.81	131.09	49.309	82.68	81.81	167.543	49.7	81.81
735	130.008	113.54	158	81.81	122.35	47.67	82.68	81.81	141.99	78.1	81.81
673.8	127.5	113.5	150	81.81	147.5	47.67	82.68	81.81	119.3	71	81.81
612.5	126.3	111.7	137	81.81	162.2	47.67	82.68	81.81	119.3	14	81.81
551.3	121.2	107.9	122	81.81	164.9	47.67	82.68	81.81	119.3	7.1	81.81
306.3	119.9	102.4	107	81.81	167.7	47.67	82.68	81.81	113.6	0	81.81

‘V’ category loads, the appliances that operate on the modern VSD technology. Examples include air-conditioners, washing machines etc.

‘D’ category loads, the loads that are inherently DC. Examples include electronics-based loads, laptop and mobile chargers, LED lights etc.

‘U’ category loads, the loads that can operate both on AC as well as DC [50,51], often termed as ‘unmediated’. All heating loads fall under this category.

**Fig 1:**
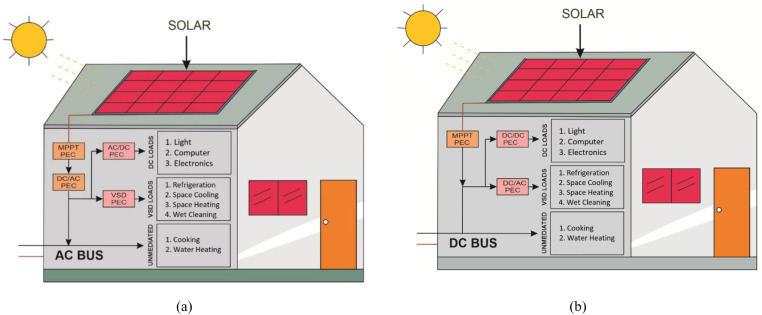
(a) AC distribution system, (b) DC distribution system.

**Fig 2:**
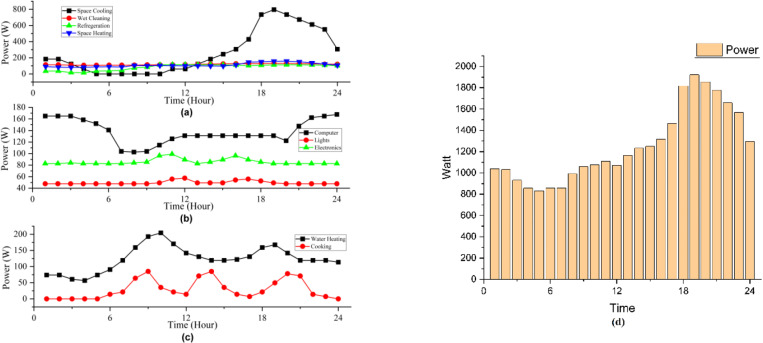
Loads characteristics (a) VSD, (b) DC and (c) Unmediated loads, and (d) Overall consumed power.

The system model for AC and DC home is presented in Fig 1 and the load profiles derived from [33] are presented in Fig 2. Fig 1 represents the AC and DC distribution systems considered for the analysis. The DC system comprises of primary distribution of 11kV, which is stepped down to secondary level to 380V, the standard DC voltage at service mains. The choice of 380V draws its support from Emerge Alliance [52], as standard DC operating voltage in distribution system. Moreover, the choice of 380V has also been highlighted in [5]. The system comprises of 10 DC transformers, each delivering power to 10 homes; making a total community of 100 DC homes. Inside each home, relevant PECs are installed to power respective loads in line with the categories defined above. The AC home is an AC analog of DC home with the difference of standard voltage level of 230V and installation of relevant PECs for respective loads, since in this case the service mains supply is AC. In addition, rooftop solar panels are installed on each residence to enhance the security and dependability of the electricity supply. AC and DC networks are similar despite the differences in conversion topologies. Fig 2 is the graphical representation of the entries of Table 1, where power consumption curves of loads falling in each category are presented with the addition of overall power consumption.

The systems presented in this paper experience transmission line losses. To accurately compare their efficiency, line resistances are taken into account for calculation of losses in transmission lines. To simplify the efficiency study, line reactance is disregarded. Moreover, it is generally accepted that the span between each pair of successive main transformers is 300 meters in length [38]. Therefore, since the line used for distribution is far shorter than the transmission line, the losses in the distribution line are so minimal in comparison to the losses in the transmission line that they may be disregarded. Additionally, this assertion is supported by [9,10,38,53]. During the modeling of the proposed system, a two-wire system is being taken into account for this study with the resistance R_ac_ double than R_dc_. The specific value of R_dc_ is 0.3006 ohms at a frequency of 60 Hz. The system’s specifications are presented in Table 2.

**Table 2:** System specification.

Parameter	Value
Oversize Ratio	10%
Photovoltaic Array	1200 Watt
Transmission Line Resistance (R_dc_)	0.3006 ohms per mile per conductor
Transmission Line Resistance (R_ac_)	0.6012 ohms per mile per conductor
Operating Frequency	50 Hz
DC Voltages	380 V
AC Voltages	230 V
Distance between Transformers	300 m

## 3. Mathematical model

The mathematical model utilized for the analysis is developed using a bottom-up approach. The mathematical model that is based on time extends from the load level all the way down to the grid level. The mathematical model calculates efficiency at the household level and also at the level of the grid or the entire distribution system, at any point in time. The load curve equations for each installed load are obtained through the utilization of the curve fitting tool. Each load has an equation of the type shown in (1).


$${\text{P}}_{\text{L}}\left(\text{t}\right){\text{=Ax}}^{\text{σ}}{\text{(t) + Bx}}^{\text{σ-1}}{\text{(t) + Cx}}^{\text{σ-2}}{\text{(t) + Dx}}^{\text{σ-3}}{\text{(t) + Ex}}^{\text{σ-4}}\text{(t) …}$$ (1)

Here, $P_{L}\left(\text{t}\right)$ is the load demand at any time (t) with coefficients A, B, C, D, E and x representing the variable in the above polynomial; and "σ" is the maximum degree of polyfit.

When a PEC is placed with a certain load, its rating is ascertained using the load curve’s maximum value. To ensure a practical and realistic approach, oversize ratio (OSR) is employed drawing its support from [12,54]. Incorporating the OSR and peak value of the load curve, (2) is devised to draw the rating of the respective load.


$${\text{R}}_{\text{PEC}}{\text{=peak (P}}_{\text{L}}\text{(t))×OSR}$$ (2)

The PEC rating is represented by $R_{PEC}$ ‘peak’ represents the maximum value of the curve. The PEC loading at any instant can be determined using (3). The loading at any instant is the ratio of the power consumed by the load at the specific instant to the rating of respective PEC, as presented in (3).


lt=PL(t)RPEC (3)

Here l(t) represents the PEC’s loading at any given time ‘t’. The realistic loading vs efficiency curve has been employed in the curve fitting tool of MATLAB for the generation of mathematical equation relating PEC loading and efficiency. The PEC’s efficiency may be mathematically expressed as (4).


$${\text{E}}_{\text{L}}{\text{=Ml}}^{\text{σ}}{\text{(t)+Nl}}^{\text{σ-1}}{\text{(t)+Ol}}^{\text{σ-2}}{\text{(t)+Pl}}^{\text{σ-3}}{\text{(t)+Ql}}^{\text{σ-4}}\text{(t)…}$$ (4)

The coefficients of the equation are M, N, O, P, and Q, and $E_{L}$ represents the PEC’s efficiency subject to the PEC’s loading at any given moment. The input power to the specific PEC, considering *i*^*th*^ load in D category driven through it, can be devised from the ratio of output load power driving the specific load to the operating efficiency at the specific instant, mathematically expressed in (5).


PDij-in(t)=PL,D(t)EL (5)

Where ${\text{P}}_{\text{Dij-in}}\text{(t)}$ represents the PEC’s input power operating the *i*^*th*^ load within the D load category in the *j*^*th*^ building. In a similar fashion, the input to all the installed PECs can be computed. To find the total power required by the *j*^*th*^ building, add up all of the input powers of the D, U, and V loads. This gives the total input power of the building available at the service mains for any instant, mathematically expressed in (6).


$${\text{P}}_{\text{j}}\left(\text{t}\right)\text{=}\left(\overset{{\text{n}}_{\text{1}}}{\underset{\text{i=1}}{\sum }}{\text{P}}_{\text{Dij-in}}\left(\text{t}\right)\right)\text{+}\left(\overset{{\text{n}}_{\text{2}}}{\underset{\text{i=1}}{\sum }}{\text{P}}_{\text{Uij-in}}\left(\text{t}\right)\right)\text{+}\left(\overset{{\text{n}}_{\text{3}}}{\underset{\text{i=1}}{\sum }}{\text{P}}_{\text{Vij-in}}\left(\text{t}\right)\right)$$ (6)

Here n_1_, n_2_, and n_3_ represent the number of appliances and ${\text{P}}_{\text{Dij-in}}$, ${\text{P}}_{\text{Uij-in}}$, and ${\text{P}}_{\text{Vij-in}}$ represent the associated power in D, U, and V, respectively.

The PECs’ efficiency placed in the *j*^*th*^ home can be computed from (7).


η(t)j=∑1nPLtPjt (7)

In this equation,η(t) is the efficiency at time (t), and ‘n’ represents the number of loads. As previously stated, the primary DC converter gives electricity to 10 homes. These homes operate as a load for the primary DC converter. The identical methodology is used to calculate the power source for a DC distribution converter at any moment, taking into account the principal transformer’s loading versus efficiency. The system’s total input is calculated by adding each input of the primary DC converters. The ratio of the total input at any moment to the total electrical energy utilized by loads of every building at that time provides the total efficiency of the system.

**Fig 3:**
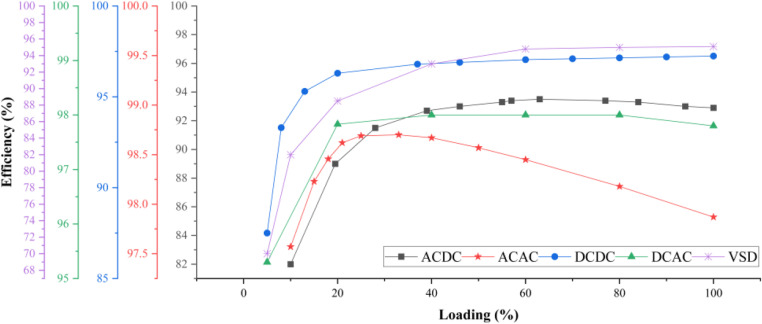
PEC efficiency w.r.t loading.

**Fig 4:**
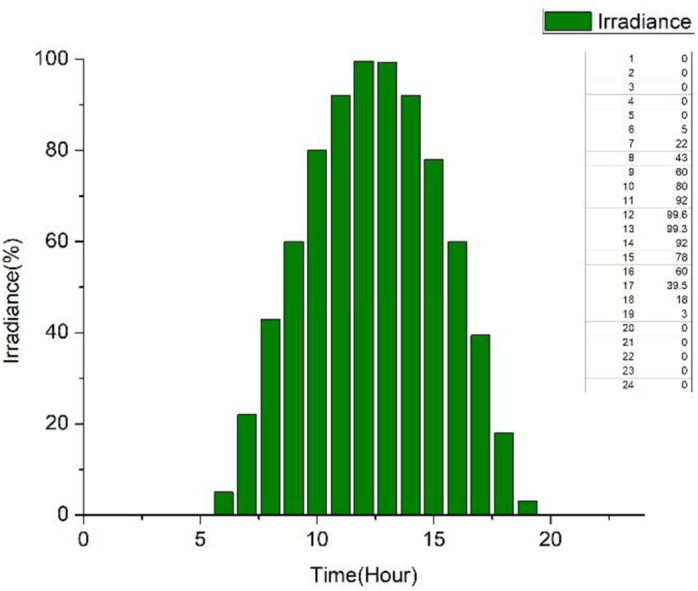
Realistic solar insolation.

The designed model employs two kinds of converters based on the application viewpoint: distribution converters and load-side converters. AC to AC and DC to DC converters are primarily employed for distribution, whereas AC to DC, VSD, and DC to AC converters are utilized at the load side to regulate the power. The following sources provide the efficiency statistics: AC to AC, DC to DC, AC to DC, DC to AC, VSD and solar irradiance available during the day [55–60]. The data is expressed as an efficiency function of the percentage loading of various converters. Fig 3 presents the loading vs efficiency curve of the installed PECs, the same curves have been employed to devise the mathematical equations which in turn are employed in the mathematical model. The realistic curve of solar insolation that is accessible during a day is presented in Fig 4, with solar power available as function of time of the day. This graph is utilized to generate expression of the solar power available to loads by using curve-fitting tool. Then maximum power from photovoltaic is obtained using MPP PEC, and the resultant electricity is transmitted to a DC to DC converter for the direct current (DC) distribution network. In the same way, a DC to AC converter processes the MPP PEC’s output for DC analogue AC systems.

The mathematical statements could be altered to accommodate the effects of solar panels installed on the roof. The sun irradiation that is accessible during a day as shown in Fig 4. The curve-fitting tool is used to calculate the energy produced by solar panels over time and its expression is represented in (8).


$${\text{P}}_{\text{j,solar}}\left(\text{t}\right){\text{= ap}}^{\text{σ}}{\text{(t) + bp}}^{\text{σ-1}}{\text{(t) + cp}}^{\text{σ-2}}{\text{(t) + dp}}^{\text{σ-3}}{\text{(t) + ep}}^{\text{σ-4}}\text{(t)…}$$ (8)

Here, ${\text{P}}_{\text{j,solar}}\left(\text{t}\right)$ denote the power generated by the solar cells at time (t), and the coefficients a, b, c, d, and e represent the equation’s parameters.

The electricity generated by PV is delivered via MPP PEC. Similar to the in-house PECs, the MPP-PEC’s rating, loading, and runtime efficiency are determined. When rooftop solar energy systems are taken into account, the home’s overall efficiency is calculated using (9).


η(t)j=∑1nPLtPj,solart+Pjt (9)

Where ${\text{η(t)}}_{\text{j}}$ represent the efficiency of *j*^*th*^ building at any instant, ${\text{P}}_{\text{L}}\left(\text{t}\right)$ represents the total load consumed and ${\text{P}}_{\text{j,solar}}\left(\text{t}\right){\text{+P}}_{\text{j}}\left(\text{t}\right)$ represents the total input power to the building at that specific instant. The system’s efficiency is evaluated using the exact identical technique as previously mentioned, i.e., incorporating the influence of ten primary DC converters and 10 homes per primary DC converter. Since AC and DC systems are analogous to each other, efficiency and the power supplied to the home and distribution converter may be computed employing the identical approach as has been done for DC system, with the exception of relevant PECs.

## 4. Results

**Fig 5:**
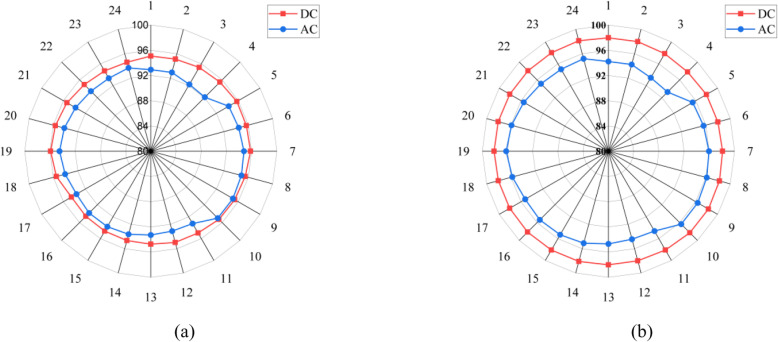
Efficiency profiles (a) Distribution network and (b) Home.

Fig 5 illustrates the efficiency profiles of AC and DC distribution networks over a day. Referring to the loading of VSD loads, which is lesser during fourth hour of the day, leads to lower operating efficiency of the installed PEC which in turn leads to a decrease in efficiency within the AC system. The effect is more pronounced in AC distribution system since the installed VSD has to go through two conversion stages adding to loss and declining the efficiency. The same pattern is also exemplified in the energy sufficiency curve. The AC efficiency curve almost keeps constant between 5^th^ to 10^th^ hour due to normal and stable operation of the VSD and DC loads. This is also due to the fact that DC and VSD loads counterbalance each other as regards to loading and in turn efficiency, keeping the overall efficiency constant. For instance, if the VSD loads are operating at low rating and the DC loads operate at high rating so as maintain a consistent effect, the same holds true for converters. Once again, the AC system efficiency decreases at 11^th^ and 12^th^ hours because the VSD loads are operating at lesser loading, the reason being same as 4^th^ hour. The DC distribution system has a relatively higher and almost constant efficiency due to the fact that all the loads are consuming power in a grouped fashion so as to balance each other demand. For example, when DC loads are low then VSD loads will take advantage to thrive and improve the efficiency of the system. Also, it is shown that the efficiency of DC distribution and in-house conversion is far higher than those for AC.

Chronically, the distribution system efficiency can be improved by setting up rooftop solar power stations which will encourage the use of DC as a potential source of current flow. The renewable energy application leads to a cutback in non-renewable energy consumption. Research in PV systems brought a revolution in to the low-carbon energy generating concept. The PV panels generate energy during the course of the day, which is sent to the MPP PEC in order to harvest the utmost power from the PV. Then, the solar power can be sent to loads via a PEC prior to the load PECs, which are DC-DC converters for DC system and DC-AC converters for AC system. In the case of DC distribution, DC loads receive power through the DC-DC converters, VSD loads receive power through the DC-AC converters, and the unmediated loads receive power directly through the distribution system. The AC-DC PEC, in contrast to the DC distribution infrastructure, is designed for powering DC appliances, whilst the VSD PEC is designed to deliver power to VSD loads. With the inclusion of solar, the priority is set to utilize the potential of installed solar PV at its maximum and in instances of load demand exceeding the installed solar power available, the loads shall be driven from grid. The highlighted circumstances apply whenever the loads receive electricity via solar energy panels.

Scenario1:

If P_Load_ > P_Solar_

Use the power from the distribution grid.

Scenario2:

If PLoad < PSolar

Use solar power.

**Fig 6:**
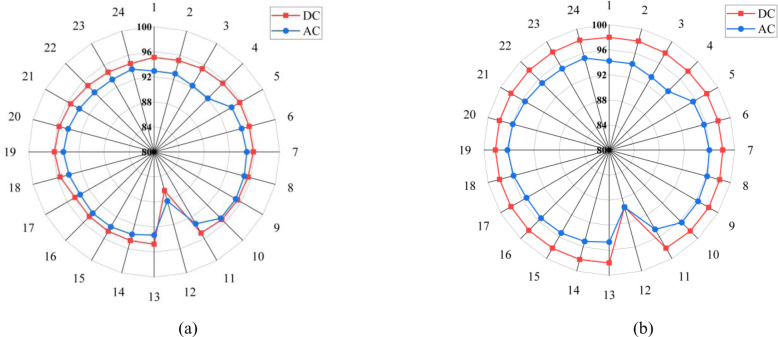
Efficiency profiles with 1.2kW solar inclusion (a) Distribution Network and (b) Home.

The impacts of integrating the already existing 1200-Watt solar system into the distribution system are illustrated in Fig 6. It clearly shows decreasing efficiency of both the building and the distribution system at 12^th^ Hour. This can be attributed to the fact that there are both solar energy and utility power at the time of the need. Since the existing efficiency assessments are carried out as a reference point, and the grid voltage becomes low, such situation results in low efficiency. Secondly, it is apparent that the efficiency of distribution system and building will stay constant as solar power meets the period when solar power is less than utility grid and energy from utility grid powers the loads.

In order to deepen the study, factors affecting the distribution system efficiency will be examined. The techniques described in this article are listed below.

1. Variation in solar capacity
2. Variation in solar capacity and efficiency of PEC
3. Variation in solar capacity

**Fig 7:**
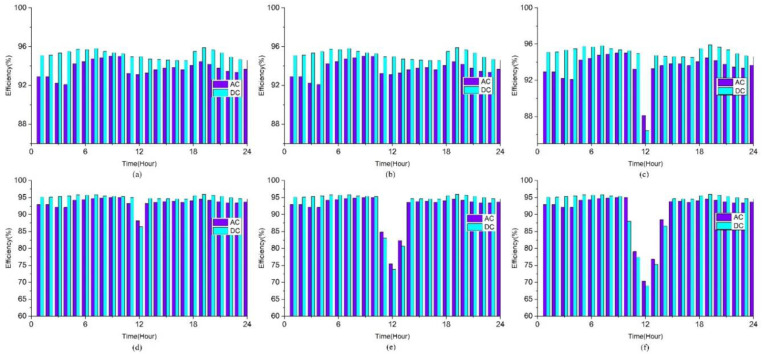
Impact of solar capacity variation on AC and DC system efficiencies for: (a) 1kW, (b) 1.1kW, (c) 1.2kW, (d) 1.3kW, (e) 1.4kW and (f) 1.5kW solar capacity.

Curiosity grew after seeing the influence of 1.2kW solar on system performance, prompting a proposal to compare the impact of solar capacity on the system’s efficiency. Furthermore, roof-top solar is positioned to deliver power to the VSD, DC, and AC loads via MPP converters. The roof-mounted solar panel has a capacity ranging from 1 kW to 1.5 kW in 100-watt increments. The results are displayed in Fig 7. It is found that regardless of the solar capacity, which is 1 kW or 1.1 kW, the distribution systems’ efficiency is constant. The operational efficiency curves of DC and AC grid systems both exhibit steep drops at 1.2 kW. Because, at that moment, the 1.2 kW of solar power supplied outweighs both utility grid and load demand. Efficiency is achieved by using the electric grid as a benchmark. As a result, efficiency suffers when the electric grid delivers less power. The efficiency of the distribution system has significantly decreased because there is now only solar power and grid electricity is not available to power the loads. Installing a 1.2kW solar panel in each of the model’s DC-Homes demonstrates an 86% distribution efficiency for the DC-based system. When a 1.5kW solar panel is added, the efficiency further drops to less than 70%. Nevertheless, a similar issue arises with the energy generated by photovoltaic panels with increased power capacity, which exhibits a rapid drop in output efficiency.

B. Variation in solar capacity and PEC’s efficiency

An efficiency performance factor or better termed as loss factor β is employed with the efficiency expression of PEC for computing the effect of variation in PEC, as depicted in (10).


$${\text{E}}_{\text{L}}{\text{= β (Ml}}^{\text{σ}}{\text{(t) + Nl}}^{\text{σ-1}}{\text{(t) + Ol}}^{\text{σ-2}}{\text{(t) + Pl}}^{\text{σ-3}}{\text{(t) + Ql}}^{\text{σ-4}}\text{(t)…)}$$ (10)

Here, σ represents the highest degree of polynomial. In this expression, β is varied from 0.96 to 1.04 with a step size of 0.02 to bring forth a change in production efficiency of PEC that would ultimately alter the efficiency of distribution system, with M, N, O, P and Q as coefficients of the polynomial. For the assessment, DC distribution and AC distribution systems have been considered with solar and without solar operation. The coefficient of β makes a difference of 2% in efficiency to the multiplication of the PEC efficiency equation.

**Fig 8:**
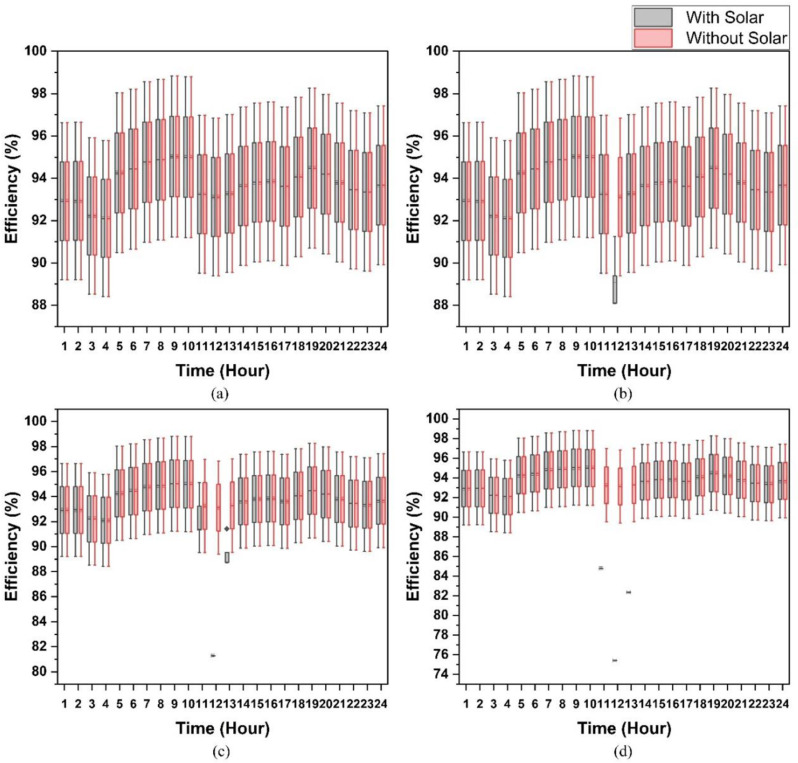
Sensitivity analysis of AC distribution system without and with variable solar capacity (a) 1.1kW, (b) 1.2kW, (c) 1.3kW, and (d) 1.4kW & variation in PEC efficiency.

**Fig 9:**
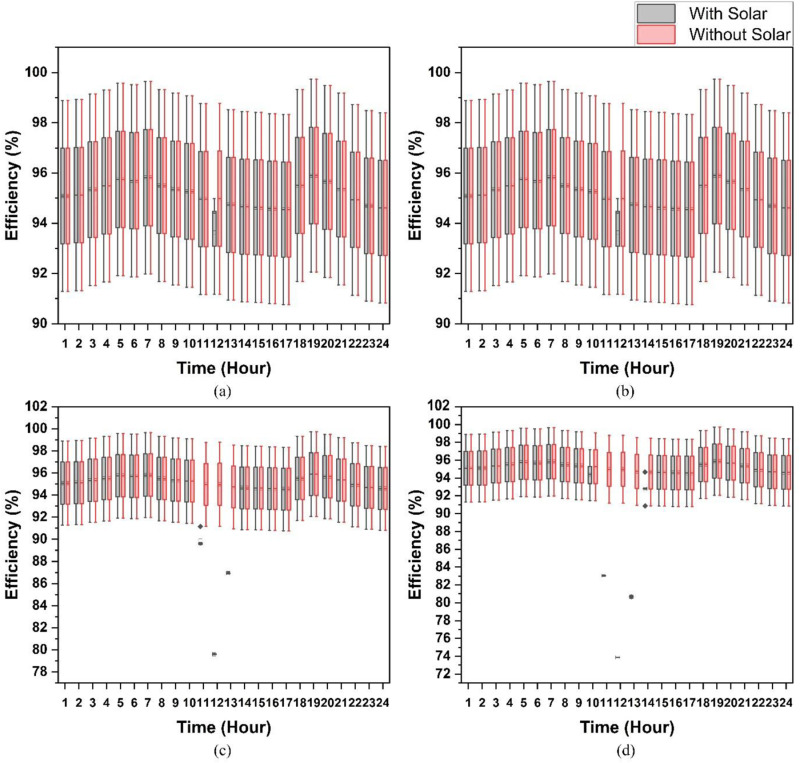
Sensitivity analysis of DC distribution system without and with variable solar capacity (a) 1.1kW, (b) 1.2kW, (c) 1.3kW, and (d) 1.4kW & variation in PEC efficiency.

In order to grasp the complexity of the AC distribution system, it is appropriate to account for the impact of the PEC efficiency variation and solar capacity variation together which enables you to know how the physical power distribution system works. Fig 8 portrays the results of our research. These graphs clearly indicate the distribution efficiencies for both systems where solar power is most plentiful and when power is supplied outside of the grid. The efficiency curves fall off drastic changes because negligible power is drawn from the electric grid for this specified duration of the day and this is when the loads are powered directly from the solar source. If solar capacity is increased, there is a significant drop in the performance of the distribution system. The rationale was the same as that of what we had already indicated. Here, the slump in efficiency is illustrated by the breadth of boxes. For a 1.5kW solar capacity, the shallow width of the box during the 12th hour is a result of the fact that the efficiency of the PEC would not be impacted, as the distribution system’s efficiency is determined by referencing the utility grid. For the case of DC distribution system, the results are shown in Fig 9. The efficiency curve has a drop for some specific hours, and it is the same as discussed above for an AC system. If a 2% change in PEC efficiency takes place, the system efficiency will rise or decrease accordingly. At β = 1, the efficiency of AC is 92.92%; 94.77% for β = 1.02; and 91.06% at β = 0.98. In the same manner, the DC’s efficiency is 95.05% for β=1, 96.98% for β=1.02, and 93.18%, for β=0.98.

**Fig 10:**
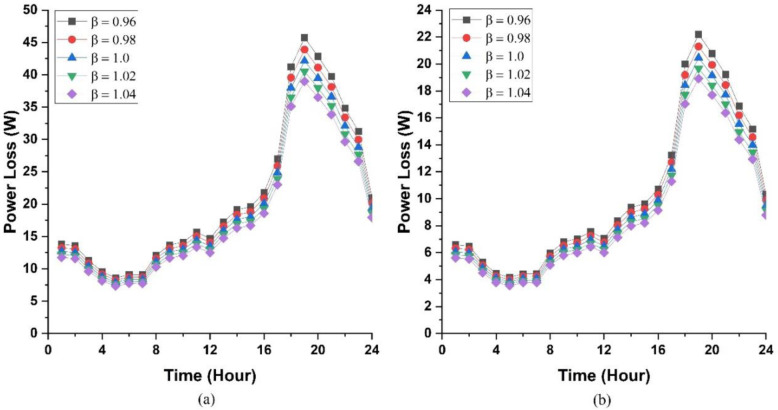
Impact of varying loss factor on power loss (a) AC distribution system and (b) DC distribution system.

To further elucidate the impact of the loss factor on power loss, a sensitivity analysis was conducted with respect to the loss factor, varying it from 0.96 to 1.04 with a step size of 0.02. The power losses in both AC and DC distribution systems were calculated over a 24-hour period, and the results are illustrated in Fig 10. These losses encompass losses in PECs and line losses. As can be seen, the power loss in both distribution systems is lower during the first six hours of the day. This is because the PECs are operating at a higher efficiency during this time frame as a result of their loading conditions. The availability of solar power and the power consumption requirements of the appliances result in higher power losses in the subsequent hours until the 20^th^ hour of the day. The scenarios described in the previous section are used to determine the loading of PECs during this period. Subsequently, the power losses decrease as a result of high operating efficiency of PECs. In comparison to the DC distribution system, the power loss in the AC distribution system is generally greater, as illustrated in Fig 10. The reason for this is that the resistance in the AC distribution system is twice as high as that in the DC distribution system, resulting in a greater power loss.

## 5. Discussion

The model in this paper is analyzed to provide an actual efficiency assessment of AC and DC distribution systems. Resistance mitigation is determined by the length of cable lines; short distribution lines demonstrate a lack of understanding of line resistance. Conversely, transmission cables are lengthy, thus we incorporated cable resistance for compensating for power loss. The efficiency analysis takes into account line losses as well as the impact of adding solar panels to the current distribution system. It is observed that the presence of solar electricity causes a significant drop in distribution system efficiency. A detailed analysis considers the variance in solar capacity and its influence on distribution efficiency.

Additionally, the distribution system sensitivity analysis showed that the system’s efficiency would change in accordance with a factor variable. If the sensitivity factor exceeds the nominal value of one, the system efficiency will correspondingly increase, while the contrary is also true. It is unveiled that the DC system transmits energy with a higher efficiency than the AC one in all sizes and configurations studied in this paper. The efficiency analysis presented in the current research effort forms a concrete base for the inclusion of DC in distribution sector. Efficiency was the factor that once wiped DC out of the power system at the time of birth of electricity and the same factor can be set a witness to the efficiency advantage of DC now. Thus, it can be regarded as the time to take the DC distribution system back into the energy network. The technological development of power electronic circuits opened the door to improve the efficiency of the systems through the provision of safety and reliability [61,62]. PECs that can provide AC to AC, AC to DC, DC to DC, DC to AC and VSD conversion, are employed in order to satisfy the load demand.

## 6. Conclusion and future work

DC turned out to be superior in the warfare of the two currents and outran AC. This thriving of DC has been because of technological advancement that has taken place in the recent past. Herein efficiency is the primary determinant of the system’s performance. Nowadays, efficiency would be the reason for DC to come back into the power system race. Various aspects were taken into account when doing the efficiency analysis. This is about the fact that there are transmission losses, PEC losses and the parasitic effects of PEC loadings. The detailed efficiency comparison between AC systems and DC systems is conducted and the DC system confirms his victory, coming out on top both in generation, then transmission, and distribution levels.

Despite fluctuating in the AC units, the DC unit efficiency maintained a relatively constant level. There are situations when DC performed better than AC because it exhibits higher PEC efficiencies at higher loading levels. On the other hand, during the day whenever the PEC efficiency was very low under poor conditions DC is relatively less efficient than AC. Additionally, we installed rooftop solar panels on the building in order to acknowledge the growing contribution of renewable power sources to electricity generation. Since our study is time-dependent, we have checked on solar power as a function of time and load fluctuations.

While rooftop solar does have fluctuating efficiency, the efficiency of residential PECs also has been modified to make the simulation system more practical. Additionally, findings revealed that the average efficiencies of both DC and AC systems were quite similar and had a positive relationship with PEC efficiencies under different percentage loading. Although AC and DC efficiencies are comparable when summed across 24 hours, DC shall surpass the AC performance during the entire day so that AC can be removed from the power distribution network completely. Therefore, we move from a single PEC to multilevel PEC to boost the generation efficiency of the entire PEC system. Smart mechanisms can be devised to switch the modules in line with the load demand. By presenting these techniques as an alternative approach to classical switching model, the efficiency of distribution system becomes more effective.

## References

[1] Hughes TP. Harold P. Brown and the executioner’s current: an incident in the ac-dc controversy. Bus Hist Rev. 1958;32(2):143–65. doi: 10.2307/3111700

[2] McPherson SS. War of the currents: thomas edison vs nikola tesla. TWENTY FIRST CENTURY BOOKS 2012.

[3] Kellow A. AC/DC: The savage tale of the first standards war. by tom mcnichol. (San Francisco, Calif.: Jossey‐Bass, 2006. Pp.198. $24.95.). The Historian. 2008;70(4):796–7. doi: 10.1111/j.1540-6563.2008.00227_29.x

[4] Rasheed A, Khan S, Gelani HE, Dastgeer F. AC vs. DC home: an efficiency comparison. 2019 international symposium on Recent Advances in Electrical Engineering (RAEE). IEEE. 2019:1–6. doi: 10.1109/raee.2019.8887064

[5] Gelani HE, Dastgeer F, Nasir M, Khan S, Guerrero JM. AC vs. DC distribution efficiency: are we on the right path?. Energies. 2021;14(13):4039. doi: 10.3390/en14134039

[6] Anees HM, Kazmi SAA, Naqvi M, Naqvi SR, Dastgeer F, Gelani HE. A mathematical model‐based approach for DC multi‐microgrid performance evaluations considering intermittent distributed energy resources, energy storage, multiple load classes, and system components variations. Energy Sci Eng. 2021;9(10):1919–34. doi: 10.1002/ese3.901

[7] Tahir H, Lee J-S, Kim R-Y. Efficiency evaluation of the microgrid for selection of common bus using copula function-based efficiency curves of the converters. Sustain Energy Technol Assess. 2021;48:101621. doi: 10.1016/j.seta.2021.101621

[8] Mughees M, Sadaf M, Erteza Gelani H, Bilal A, Saeed F, Chowdhury MdS, et al. Comparison of efficiency-based optimal load distribution for modular ssts with biologically inspired optimization algorithms. Electronics. 2022;11(13):1988. doi: 10.3390/electronics11131988

[9] Tahir H, Gelani HE, Saleem M, Hussain A. Efficiency and reliability assessment-based selection of the optimal common bus in hub-stations. Electronics. 2023;12(16):3411. doi: 10.3390/electronics12163411

[10] Dastgeer F, Gelani HE, Anees HM, Paracha ZJ, Kalam A. Analyses of efficiency/energy-savings of DC power distribution systems/microgrids: Past, present and future. Int J Electr Power Energy Syst. 2019;104:89–100. doi: 10.1016/j.ijepes.2018.06.057

[11] Souza Junior MET, Freitas LCG. Power electronics for modern sustainable power systems: Distributed generation, microgrids and smart grids—a review. Sustainability. 2022;14(6):3597. doi: 10.3390/su14063597

[12] Fisher AN, Stinson DA, Kalajdzic A, Dupuis HE, Lowey EE, Desgrosseilliers E, et al. “A Recipe for Disaster?”: female-breadwinner relationships threaten heterosexual scripts. Sex Roles. 2025;91(3):16. doi: 10.1007/s11199-025-01560-y 39990977 PMC11839713

[13] Zolfaghari M, Gharehpetian GB, Shafie-khah M, Catalão JPS. Comprehensive review on the strategies for controlling the interconnection of AC and DC microgrids. Int J Electr Power Energy Syst. 2022;136:107742. doi: 10.1016/j.ijepes.2021.107742

[14] Agundis Tinajero GD, Nasir M, Vasquez JC, Guerrero JM. Comprehensive power flow modelling of hierarchically controlled AC/DC hybrid islanded microgrids. Int. J. Electr. Power Energy Syst. 2021;127:106629. doi: 10.1016/j.ijepes.2020.106629

[15] Hansson BON. Submarine cable for 100-kv D-C power transmission. Electr Eng. 1954;73(7):602–602. doi: 10.1109/ee.1954.6438864

[16] Seyezhai R, Karuppuchamy S, Ashok Kumar L. Recent trends in renewable energy sources and power conversion. Springer Proc Energy. Springer Singapore; 2021. doi: 10.1007/978-981-16-0669-4

[17] DaneshvarDehnavi S, Negri CA, Giesselmann MG, Bayne SB, Wollenberg B. Can 100% renewable power system be successfully built?. Renew Energy. 2021;177:715–22. doi: 10.1016/j.renene.2021.06.002

[18] Patel MR, Beik O. Wind and solar power systems. CRC Press. 2021. doi: 10.1201/9781003042952

[19] Elkasem AHA, Khamies M, Magdy G, Taha IBM, Kamel S. Frequency stability of ac/dc interconnected power systems with wind energy using arithmetic optimization algorithm-based fuzzy-pid controller. Sustainability. 2021;13(21):12095. doi: 10.3390/su132112095

[20] Ali S, Zheng Z, Aillerie M, Sawicki J-P, Péra M-C, Hissel D. A review of DC microgrid energy management systems dedicated to residential applications. Energies. 2021;14(14):4308. doi: 10.3390/en14144308

[21] Dastgeer F, Muhammad Anees H, Gelani HE, Amjad K, Rameez Javeed M. A basic mathematical testbed for energy efficiency analyses of DC power distribution systems/microgrids. Bulletin EEI. 2021;10(3). doi: 10.11591/eei.v10i3.2775

[22] Ben Abdeljawed H, El Amraoui L. Simulation and rapid control prototyping of DC powered universal motors speed control: towards an efficient operation in future DC homes. Eng Sci Technol Int J. 2022;34:101092. doi: 10.1016/j.jestch.2021.101092

[23] Dragicevic T, Wheeler P, Blaabjerg F. DC distribution systems and microgrids. Institution of Engineering and Technology. 2018. doi: 10.1049/pbpo115e

[24] Charadi S, Chaibi Y, Redouane A, Allouhi A, El Hasnaoui A, Mahmoudi H. Efficiency and energy‐loss analysis for hybridAC/DCdistribution systems and microgrids: a review. Int Trans Electr Energ Syst. 2021;31(12). doi: 10.1002/2050-7038.13203

[25] Habibi S, Rahimi R, Shamsi P, Ferdowsi M. Efficiency assessment of a residential DC nanogrid with low and high distribution voltages using realistic data. 2021 IEEE GreenTech Conf. 2021:574–9. doi: 10.1109/greentech48523.2021.00096

[26] Boeke U, Wendt M. DC power grids for buildings. 2015 IEEE 1st Int. Conf. DC Microgrids (ICDCM). 2015. doi: 10.1109/icdcm.2015.7152040

[27] Fregosi D, Ravula S, Brhlik D, Saussele J, Frank S, Bonnema E, et al. A comparative study of DC and AC microgrids in commercial buildings across different climates and operating profiles. 2015 IEEE 1st Int Conf DC Microgrids (ICDCM). 2015. doi: 10.1109/icdcm.2015.7152031

[28] Goikoetxea A, Canales JM, Sanchez R, Zumeta P. DC versus AC in residential buildings: efficiency comparison. Eurocon 2013. 2013:1–5. doi: 10.1109/eurocon.2013.6625162

[29] Sirsi R, Prasad S, Sonawane A, Lokhande A. Efficiency comparison of AC distribution system and DC distribution system in microgrid. 2016 Int Conf Energy Efficient Technol Sustain (ICEETS). 2016:325–9. doi: 10.1109/iceets.2016.7583774

[30] Amin M, Arafat Y, Lundberg S, Mangold S. Low voltage DC distribution system compared with 230 V AC. 2011 IEEE Electr Power Energy Conf. 2011:340–5. doi: 10.1109/epec.2011.6070222

[31] Sirsi R, Ambekar Y. Efficiency of DC microgrid on DC distribution system. 2015 IEEE Innov. Smart Grid Technol. - Asia (ISGT ASIA). 2015:1–6. doi: 10.1109/isgt-asia.2015.7387055

[32] Chauhan RK, Rajpurohit BS. DC distribution system for energy efficient buildings. 2014 18th Nat Power Syst Conf. (NPSC). 2014:1–6. doi: 10.1109/npsc.2014.7103813

[33] U.S. Energy Information Administration. Household electricity consumption. 2015. https://www.eia.gov/consumption/residential/data/2015/c&e/pdf/ce5.1a.pdf

[34] Gelani H, Dastgeer F, Siraj K, Nasir M, Niazi K, Yang Y. Efficiency comparison of AC and DC distribution networks for modern residential localities. Appl Sci. 2019;9(3):582. doi: 10.3390/app9030582

[35] Nasir M, Khan HA. Solar photovoltaic integrated building scale hybrid AC/DC microgrid. 5th IET Int Conf Renewable Power Generat. (RPG) 2016. 2016. doi: 10.1049/cp.2016.0528

[36] Brenguier J, Vallet M, Vaillant F. Efficiency gap between AC and DC electrical power distribution system. 2016 IEEE/IAS 52nd Ind. Commercial Power Syst. Tech. Conf. (I&CPS). 2016. doi: 10.1109/icps.2016.7490224

[37] Gerber DL, Vossos V, Feng W, Marnay C, Nordman B, Brown R. A simulation-based efficiency comparison of AC and DC power distribution networks in commercial buildings. Appl Energy. 2018;210:1167–87. doi: 10.1016/j.apenergy.2017.05.179

[38] Gelani HE, Dastgeer F. Efficiency analyses of a DC residential power distribution system for the modern home. AECE. 2015;15(1):135–42. doi: 10.4316/aece.2015.01018

[39] Manandhar U, Ukil A, Kiat Jonathan TK. Efficiency comparison of DC and AC microgrid. 2015 IEEE Innov Smart Grid Technol - Asia (ISGT ASIA). 2015. doi: 10.1109/isgt-asia.2015.7387051

[40] Nilsson D, Sannino A. Efficiency analysis of low- and medium-voltage dc distribution systems. IEEE Power Eng Soc Gen Meet. 2004. 2004;2:2316–22. doi: 10.1109/pes.2004.1373299

[41] Siraj K, Khan HA. DC distribution for residential power networks—a framework to analyze the impact of voltage levels on energy efficiency. Energy Rep. 2020;6:944–51. doi: 10.1016/j.egyr.2020.04.018

[42] Liu Z, Li M. Research on energy efficiency of DC distribution system. AASRI Procedia. 2014;7:68–74. doi: 10.1016/j.aasri.2014.05.031

[43] Weiss R, Ott L, Boeke U. Energy efficient low-voltage DC-grids for commercial buildings. 2015 IEEE 1st Int Conf DC Microgrids (ICDCM). 2015. doi: 10.1109/icdcm.2015.7152030

[44] Hammerstrom DJ. AC versus DC Distribution systemsdid we get it right?. 2007 IEEE Power Eng Soc Gen Meet. 2007. doi: 10.1109/pes.2007.386130

[45] Starke M, Tolbert LM, Ozpineci B. AC vs. DC distribution: a loss comparison. 2008 IEEE/PES Transm Distrib Conf Expos. 2008. doi: 10.1109/tdc.2008.4517256

[46] Atia HR, Shakya A, Tandukar P, Tamrakar U, Hansen TM, Tonkoski R. Efficiency analysis of AC coupled and DC coupled microgrids considering load profile variations. 2016 IEEE Int Conf Electro Inf Technol (EIT). 2016:0695–9. doi: 10.1109/eit.2016.7535324

[47] Dastgeer F, Gelani HE. A Comparative analysis of system efficiency for AC and DC residential power distribution paradigms. Energy Build. 2017;138:648–54. doi: 10.1016/j.enbuild.2016.12.077

[48] Glasgo B, Azevedo IL, Hendrickson C. How much electricity can we save by using direct current circuits in homes? Understanding the potential for electricity savings and assessing feasibility of a transition towards DC powered buildings. Appl Energy. 2016;180:66–75. doi: 10.1016/j.apenergy.2016.07.036

[49] Vossos V, Garbesi K, Shen H. Energy savings from direct-DC in U.S. residential buildings. Energy Build. 2014;68:223–31. doi: 10.1016/j.enbuild.2013.09.009

[50] Erteza Gelani H, Dastgeer F, Ali Shah SA, Saeed F, Hassan Yousuf M, Afzal HMW, et al. Comparative efficiency and sensitivity analysis of AC and DC power distribution paradigms for residential localities. Sustainability. 2022;14(13):8220. doi: 10.3390/su14138220

[51] Ahmad F, Dastgeer F, Gelani HE, Khan S, Nasir M. Comparative analyses of residential building efficiency for AC and DC distribution networks. Bull Pol Acad Sci Tech Sci. 2021:136732–136732. doi: 10.24425/bpasts.2021.136732

[52] EMerge Alliance. EMerge Alliance—Hybrid AC/DC & DC microgrid standards & information. 2024. https://www.emergealliance.org/

[53] Stevenson W, Grainger J. Power system analysis. MC Graw Hill; 1994. https://www.mheducation.com/highered/product/power-system-analysis-stevenson-grainger/M9780070612938.html

[54] Shao S, Pipattanasomporn M, Rahman S. Challenges of PHEV penetration to the residential distribution network. 2009 IEEE Power Energy Soc Gen Meet. 2009:1–8. doi: 10.1109/pes.2009.5275806

[55] Malamaki K-ND, Demoulias CS. Minimization of electrical losses in two-axis tracking pv systems. IEEE Trans Power Delivery. 2013;28(4):2445–55. doi: 10.1109/tpwrd.2013.2272405

[56] Kazimierczuk M, Ayachit A, Saini D. Average current‐mode control of DC‐DC power converters. 2022. doi: 10.1002/9781119525592

[57] De Almeida AT, Ferreira FJTE, Fong JAC. Standards for efficiency of electric motors. IEEE Ind Appl Mag. 2011;17(1):12–9. doi: 10.1109/mias.2010.939427

[58] National Renewable Energy Laboratory. Solar resource data and tools. 2011. https://www.nrel.gov/grid/solar-resource/renewable-resource-data.html

[59] Hartmann M, Ertl H, Kolar JW. EMI filter design for a 1 MHz, 10 kW three-phase/level PWM rectifier. IEEE Trans Power Electron. 2011;26(4):1192–204. doi: 10.1109/tpel.2010.2070520

[60] Bragard M, Soltau N, De Doncker RW, Schmiegel A. Design and implementation of a 5 kW photovoltaic system with li-ion battery and additional DC-DC converter. 2010 IEEE Energy Convers Congr Expos. 2010. doi: 10.1109/ecce.2010.5618220

[61] Alcaide AM, Buticchi G, Chub A, Dalessandro L. Design and control for high-reliability power electronics: state-of-the-art and future trends. IEEE J Emerg Sel Top Ind Electron. 2024;5(1):50–61. doi: 10.1109/jestie.2023.3287513

[62] Sandelic M, Peyghami S, Sangwongwanich A, Blaabjerg F. Reliability aspects in microgrid design and planning: status and power electronics-induced challenges. Renew. Sustain. Energy Rev. 2022;159:112127. doi: 10.1016/j.rser.2022.112127

